# Dr. S. S. Gray, Memphis. Tenn

**Published:** 1862-08

**Authors:** 


### OBITUARY.

DR. S. C. GRAY, a dentist, formerly of this city, but recently of Memphis, Tenn., died almost one year ago, of typhoid fever.

The Doctor went a few years ago from here to Memphis, and succeeded in establishing himself in a good practice. He was a gentleman in every sense, and a good operator, one who had the esteem of his professional brethren wherever known. The closure of communication between this and that part of the country prevented our obtaining a knowledge of his demise until just recently. T.

